# Correlation between quantitative analysis of wall shear stress and intima-media thickness in atherosclerosis development in carotid arteries

**DOI:** 10.1186/s12938-017-0425-9

**Published:** 2017-12-06

**Authors:** Bo Zhang, Junyi Gu, Ming Qian, Lili Niu, Hui Zhou, Dhanjoo Ghista

**Affiliations:** 1 Department of Ultrasound in Medicine, Shanghai East Hospital, Tongji University School of Medicine, Shanghai, 200120 China; 2 Paul C. Lauterbur Research Center for Biomedical Imaging, Shenzhen Institutes of Advanced Technology, Chinese Academy of Sciences, Shenzhen, 518055 China; 3 University 2020 Foundation, Northborough, MA 01532 USA

**Keywords:** Carotid artery, Fibrous plaques, Wall shear stress, Intima-media thickness, Receiver operator characteristic

## Abstract

**Background:**

This paper presents quantitative analysis of blood flow shear stress by measuring the carotid arterial wall shear stress (WSS) and the intima-media thickness (IMT) of experimental rabbits fed with high-fat feedstuff on a weekly basis in order to cause atherosclerosis.

**Methods:**

This study is based on establishing an atherosclerosis model of high-fat rabbits, and measuring the rabbits’ common carotid arterial WSS of the experimental group and control group on a weekly basis. Detailed analysis was performed by using WSS quantification.

**Results:**

We have demonstrated small significant difference of rabbit carotid artery WSS between the experimental group and the control group (P<0.01) from the 1st week onwards, while the IMT of experimental group had larger differences from 5th week compared with the control group (P<0.05). Next, we have shown that with increasing blood lipids, the rabbit carotid artery shear stress decreases and the rabbit carotid artery IMT goes up. The decrease of shear stress appears before the start of IMT growth. Furthermore, our receiver operator characteristic (ROC) curve analysis showed that when the mean value of shear stress is 1.198 dyne/cm^2^, the rabbit common carotid atherosclerosis fatty streaks sensitivity is 89.8%, and the specificity is 81.3%. The area under the ROC curve is 0.9283.

**Conclusions:**

All these data goes to show that WSS decreasing to 1.198 dyne/cm^2^ can be used as an indicator that rabbit common carotid artery comes into the period of fibrous plaques. In conclusion, our study is able to find and confirm that the decrease of the arterial WSS can predict the occurrence of atherosclerosis earlier, and offer help for positive clinical intervention.

## Background

Epidemiological investigations have shown that cardiovascular and cerebrovascular diseases are the major lethal factors affecting people around the world. In 2008, the national monitoring data from The National Centers for Disease Control and Prevention showed that cardiovascular and cerebrovascular diseases mortality in China is 229/100 thousands [1], and atherosclerosis is a big cause of cardiovascular and cerebrovascular diseases. In terms of cerebral infarction, the primary cause is atherosclerosis [2].

The formation of atherosclerosis is a long and complicated process. Pathology shows that atherosclerosis is generally divided into four periods: (1) fatty streaks, (2) fibrous plaque, (3) atheromatous plaque, and (4) complicated lesions or secondary changes [3]. Early atherosclerosis refers to the clinical precancerous lesion of atherosclerosis (Preclinical Atherosclerosis, PCA), which means that the patients has evidence of atherosclerosis, but there are no specific clinical symptoms of atherosclerotic stenosis caused by arterial atherosclerosis [4].

The endothelium lining the cardiovascular system is highly sensitive to hemodynamic shear stresses that act on the vessel luminal surface in the direction of blood flow. Physiological variations of shear stress regulate changes in structural-wall remodeling, and are associated with susceptibility to atherosclerosis. Hence, identification and diagnosis of atherosclerosis in its early stage and then doing timely intervention can reduce (i) the occurrence rate of myocardial infarction and cerebral infarction after the expansion of plaques, and (ii) the consequences of interventional therapies such as angioplasty, bypass grafts, and deployment of stents. Thus medical cost can also be reduced, which has significant social and economic value.

Noninvasive assessment of atherosclerosis, such as intimal wall thickening and plaque formation, is routinely available using a variety of imaging techniques. The most common clinical detection of atherosclerosis is to measure the intima-media thickness (IMT) of common carotid artery by using ultrasonic imaging. By measuring the IMT, we can judge whether there is atherosclerosis and even atherosclerotic plaques [5, 6]. However, some scholars have suggested that IMT provides only a limited indication for early diagnosis of atherosclerosis and predicting cardiovascular and cerebrovascular diseases [7, 8].

Other methods for the clinical diagnosis of atherosclerosis mainly include digital subtraction angiography, magnetic resonance angiography, and computed tomography (CT) angiography. The above technologies are mainly based on the vascular morphological changes and hemodynamics, to observe vascular intima-media thickness, plaques, degree of luminal stenosis, and the blood flow velocity and flow rate, etc. Because in the early stage of atherosclerosis, there is no apparent vascular morphology change or hemodynamic changes, the above technologies cannot effectively predicate the early stage of atherosclerosis.

Vessel segments with low wall shear stress or highly oscillatory wall shear stress appear to be at the highest risk for development of atherosclerosis. A large number of experiments have shown that the reduction of vessel wall shear stress (WSS) has a close relationship with the incidence of atherosclerosis [9, 10]. WSS change can directly affect the morphology and function of the vascular endothelium, and stimulate the migration and proliferation of vascular endothelial smooth muscle cells and mononuclear cells [11]. Low or unstable changing WSS is an index of the occurrence and development of vascular atherosclerosis [12]. It is increasingly valued as an indicator to evaluate hemodynamic changes that are closely related to atherosclerosis [13, 14].

In this study, we have used WSS quantitative analysis software for carrying out WSS quantitative analysis [15]. This method can accurately show WSS changes at different spatial locations. It is a kind of convenient and noninvasive vascular WSS analysis tool. For this research, we have established an atherosclerosis model of high-fat rabbits, and have weekly measured the rabbits’ common carotid arterial WSS of the experimental group and control group by using WSS quantitative analysis. Then, we have computed ROC curves when the pathological histology prompts that the common carotid arteries of the experimental rabbits are at the stage of fatty streaks and arterial fiber plaques. In particular, we made sure of the WSS threshold for atherosclerosis stages, and of the sensitivity and specificity of this threshold.

Although now it is widely accepted that low or unstable changing WSS is a high-risk signal in the development of vascular atherosclerosis, still there are only a few studies on the correlation between the WSS specific data and atherosclerosis. Hence, this study aims to find out the correlation between WSS and atherosclerosis, by observing the dynamic changes in the arterial blood wall pathological histology.

## Materials and methods

### Experimental animals and grouping

In our experimental study, we employed a total of 60 healthy white male New Zealand rabbits (provided by Shanghai Tongji University Animal Laboratory), which are approximately 10 weeks old, and weighed 2–2.5 kg. By use of a randomized method, they were divided into two groups: 20 in normal control group and in the experimental group. Ethics on animal experiments were approved by the institutional board of Tongji University.

### Instruments

The Philips IE33 Diasonograph (Philips Medical Systems, Andover, MA, USA) as well as high frequency probe L15-7 (Philips Medical Systems, Andover, MA, USA) were used in our study.

### Pharmaceutical application

We applied Atropine Sulfate injection, 0.5 mg/ml (developed by Shanghai Wellhope Pharmaceutical Co. Ltd., and approved by H31021172). Then, for Ketamine hydrochloride injection, we implemented 2 ml at 0.1 g (by Jiangsu Hengrui Pharmaceutical Co. Ltd., and approved by H32022820).

### Animal models

Experimental rabbits were fed with high-fat feedstuff, which was bought from Trophy Feedstuff Technology Co. Ltd. (where feedstuff code is TP2R118), and caused to develop atherosclerosis artificially. These New Zealand rabbits in the experimental group were fed with the feedstuff at 50 g/kg/days once every 12 h, and allowed to drink water with no restraint for a total of 10 weeks. The temperature of breeding environment was controlled at around 15 °C, and the rooms are kept ventilated and clean in accordance to animal ethnics.

### Blood test and rabbit carotid artery specimen collection

#### Blood test

We extracted phlebotomize from the rabbit’s ear section once a week. Then, we used the automatic biochemical analyzer enzyme standard method to detect the serum total cholesterol (TC), triglyceride (TG), high-density lipoprotein (HDL) and low density lipoprotein (LDL).

#### Rabbit carotid artery specimens collection

Every two weeks, we sacrificed 8 rabbits in the experimental group and 4 rabbits in the control group by the air embolism method. We surgically removed the rabbits’ common carotid arteries and marked the proximal parts with thick thread ties. The extracted common carotid arteries were fixed in 10% formaldehyde solution, and then we applied and examined the paraffin section and HE stain. Next, we performed histological observation under light, and recorded the histologic appearances of the arterial wall during different periods.

### Rabbit carotid artery IMT measurements

We implemented the experimental rabbit intramuscular anesthesia with ketamine hydrochloride (22 mg/kg) and atropine sulfate (70 μg/kg) mixture [16]. We used Philips IE33 diasonograph, L15-7 linear array transverse section to scan the rabbit carotid arteries, about 1–2 cm below the mandibular angle plane, of both the experimental group and the control group, to collect 2 days images of the common carotid artery. By partial enlargement and adjusting the gain, the rabbit carotid artery wall intima-media is clearly shown. Then, we vertically placed the vascular wall of the carotid artery and measured the IMT value.

### Quantitative analysis of shear stress

#### Principle of quantitative analysis of wall shear stress

**Fig. 1:**
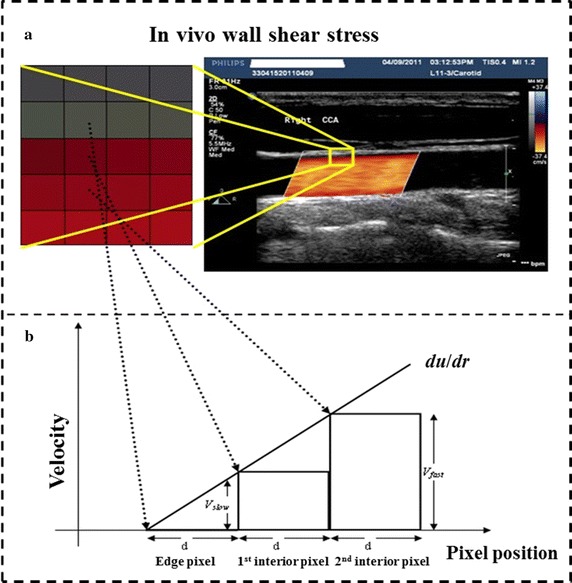
Principle of quantitative analysis of shear stress based on **a** ultrasound imaging of artery, and **b** velocity gradient used in the Hagen–Poiseuille formula

In Fig. 1, part A is a Color Doppler blood flow diagram (or Color Doppler flow Imaging, CDFI). The magnified image on the left side is the pixel magnified image of the yellow box area in the vascular wall. Part B illustrates the shear stress calculation principle. In the Doppler blood flow image, the luminal border pixels are shown by grayscale pixels, and so the speed of the boundary pixels is zero. The blood flow near the border is shown by colored pixels. The brightness of the colored pixel is proportional to the blood flow velocity. The following definitions are made: *V*_*slow*_ is the speed of blood flow pixels of Doppler blood flow velocity parallel to the wall and very close to the vessel wall. *V*_*fast*_ is the speed of next layer of blood flow pixels of Doppler blood flow velocity parallel to the wall. The distance between two adjacent layers of pixels is constant, and defined as *d*. In the Doppler blood flowing image, the change in axial velocity in the radial direction, *du*/*dr*, is based on the equation:

1 $$\frac{du}{dr} = \frac{{V_{fast} - V_{slow} }}{d},$$

which can be the approximation of the blood WSS:

2 $$\tau_{w} = \mu \gamma_{w} = \mu \frac{du}{dr}|r = wall,$$

where *μ* is the fluid viscosity value.

Traditionally, the Hagen–Poiseuille formula (see below Eqs. 3 and 4) is applied to determine the WSS, by measuring the diameter of the vascular cavity, and the blood flow rate or the maximum flow velocity at the flow observation point. Due to its simplicity, the Hagen–Poiseuille formula can be used clinically, even though it is based on fluid flow in ideal conditions. The human artery blood vessel does not assume a standard circular cross-section, and the WSS on the vascular wall is affected by different factors like blood flow, blood pressure, tube wall geometry and intima-media thickness, etc. Nevertheless, the WSS values obtained from the Hagen–Poiseuille formula can adequately reflect WSS changes in blood vessels due to flow rate, fluid viscosity and vessel radius [17], as given by the following equations:

3 $$\tau_{w} = \frac{4\mu Q}{{\pi R^{3} }}.$$

where in *τ*_*w*_ is wall shear stress, *Q* is flow rate, *μ* is fluid viscosity *R* is vessel radius, and *u*_*M*_ is the maximum velocity of the fluid (at the center of the artery).

For the fluid shear stress, we have

4 $$\tau = \frac{{2\mu u_{M} }}{{\pi R^{3} }}.$$

#### Image acquisition for wall shear stress quantitative analysis

We used the Philips IE33 diasonograph, L15-7 linear array probe for scanning the rabbit carotid arteries in the longitudinal section, and we ensured that the ultrasonic cross-section passes through the center axis of the blood vessels. The acoustic beam was at 60° angle to the common carotid artery. We adjusted (i) the speed range to make the lumen full of blood flow without aliasing, and also (ii) the sampling frame range, in order to keep the Doppler graphics frame frequency between 20–30 frames. Then, we collected the Doppler blood flow images of the common carotid artery, about 1–2 cm below the mandibular angle plane, in both the experimental group and the control group. The images were saved in the DICOM format. Finally, we employed the software for quantitative analysis of shear stress, as follows:

1. Obtain the Doppler speed range.
2. Adjust the angle of image display. Because it is difficult to keep the blood vessel level in the ultrasound images in order to facilitate calculating the axial velocity gradient perpendicular to the blood flow, it becomes necessary to adjust the flow in the blood vessels in the image into a horizontal position.
3. Select the area-of-interest used for determining the wall shear stress.
4. Weed out the gray-scale images used for showing the vascular wall.
5. Transform the color images that represent Doppler blood flow velocity into velocity data, according to the maximum speed and according to the color indication range.
6. Blood viscosity is set to 3 cp.
7. Draw the shear stress space distribution.
8. Show the shear stress distribution, as required.
9. Analysis of shear stress data.
10. Results of images are saved in digital image format, and the data values are saved in a database.

### Statistical processing

The SAS9.3 software is used for statistical analysis. The measurement data is described based on $\bar{x} \pm s$. Comparison among groups uses t test. Diagnostic efficiency is shown by sensitivity and specificity. We build an ROC curve of rabbit’s common carotid arterial WSS, and calculate the area under the curve to evaluate the diagnosis. A P < 0.05 means the difference has a statistical significance.

## Experimental results

### Histopathologic examination

#### Rabbit carotid artery histology of the control group

**Fig. 2:**
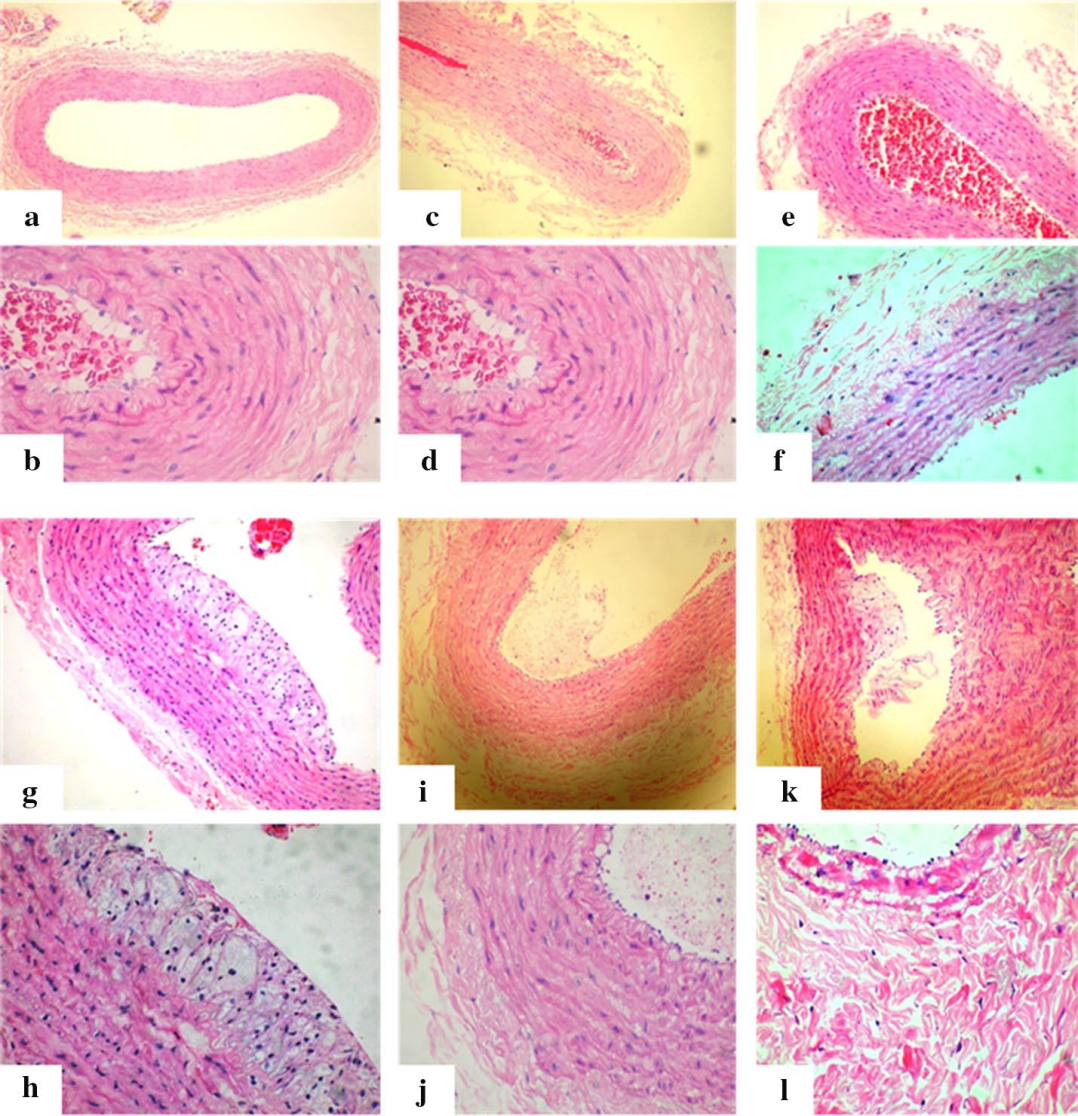
Rabbit carotid artery histology of the control group with microscopic low power lens (**a**) and high power lens (**b**). Rabbit carotid artery histology of the experimental group at 2 weeks (**c**, **d**), 4 weeks (**e**, **f**), 6 weeks (**g**, **h**), 8 weeks (**i**, **j**), and 10 weeks (**k**, **l**)

Based on observation, the structures of arterial intima, tunica media and tunica externa are complete. The internal elastic membrane is continuous. No intimal thickening and no foam cells beneath intima are seen (Fig. 2a, b).

#### Carotid artery pathology of the experimental rabbits

For rabbit carotid artery histology of the experimental group (after 2 weeks), the structures of arterial intima, tunica media and tunica externa are complete. The internal elastic membrane is continuous. No intimal thickening and no foam cells beneath intima are seen (Fig. 2c, d).

For the rabbit carotid artery histology of the experimental group (after 4 weeks), the structures of arterial intima, tunica media and tunica externa are complete. The internal elastic membrane is continuous. No intimal thickening and no foam cells beneath intima are seen (Fig. 2e, f).

For the rabbit’s common carotid artery histology of the experimental group (after 6 weeks), we see that foam cells are gathered under the endothelial cells of the artery intimal surface, convex to the lumen, and the formation of some fatty streaks. There are more extracellular matrixes at the edges. Smooth muscle cells and collagen fiber hyperplasia are not obvious. The fatty streaks period is shown in Fig. 2g, h.

For carotid artery pathology of experimental rabbits (after 8 weeks), we can see a large number of foam cells gathered under the endothelial cells. Plaques are formed. The arterial wall’s thickness is uneven, and some parts of the walls have become thicker. Smooth muscle cells are seen to proliferate. Figure 2i, j shows the fibrous plaques formation period.

For carotid artery pathology of experimental rabbits (following 10 weeks), a large number of foam cells are seen to gather under the endothelial cells and subsequently, plaques are formed. The arterial wall thickness is seen to be uneven and some parts of the wall have become thicker. We can also see smooth muscle cells and collagen fiber hyperplasia in the pathology. Figure 2k, l shows the dense fibrous plaque formation period.

### Blood lipid test and analysis

#### Quantitative value of rabbit serum total cholesterol

There are seen to be statistical differences in *t* test between the two groups from the first week; the serum cholesterol level of the experimental rabbits is higher than that of the control group from the first week (as depicted in Table 1).

**Table 1:** Serum total cholesterol test results between the experimental group and the control group (mmol/L)

Group	Week 1	Week 2	Week 3	Week 4	Week 5	Week 6	Week 7	Week 8	Week 9	Week 10
Exp.	14.45 ± 1.99	27.52 ± 3.96	37.40 ± 7.30	44.92 ± 11.47	48.38 ± 12.21	48.52 ± 9.96	47.19 ± 9.35	50.46 ± 10.78	46.04 ± 9.89	52.77 ± 11.25
Control	1.17 ± 0.40	1.29 ± 0.42	1.35 ± 0.45	1.29 ± 0.43	1.37 ± 0.38	1.37 ± 0.43	1.19 ± 0.37	1.32 ± 0.41	1.25 ± 0.40	1.40 ± 0.43
P value	0.0002	0.0001	0.0001	0.0001	0.0001	0.0001	0.0001	0.0001	0.0001	0.0001

Conduct *t* test on the rabbit serum cholesterol values of the experimental group and the control group. P values < 0.05 from the 1st week. See statistical differences in the two groups

#### Rabbit serum low density lipoprotein

There are statistical differences in *t*-test between the two groups from the first week. It is seen that the serum low density lipoprotein level of the experimental rabbits is higher than that of the control group from the first week (as depicted in Table 2).

**Table 2:** Serum low density lipoprotein test results between the experimental group and the control group (mmol/L)

Group	Week 1	Week 2	Week 3	Week 4	Week 5	Week 6	Week 7	Week 8	Week 9	Week 10
Exp.	13.02 ± 1.89	25.31 ± 3.71	34.06 ± 6.18	39.63 ± 9.99	43.85 ± 9.98	44.96 ± 9.15	43.76 ± 8.77	47.94 ± 9.35	43.69 ± 7.07	50.69 ± 7.47
Control	0.41 ± 0.67	0.43 ± 0.57	0.42 ± 0.60	0.45 ± 0.63	0.42 ± 0.60	0.45 ± 0.58	0.46 ± 0.63	0.50 ± 0.53	0.49 ± 0.58	0.65 ± 0.64
P value	0.0002	0.0001	0.0001	0.0001	0.0001	0.0001	0.0001	0.0002	0.0003	0.0001

Conduct *t* test on the rabbit serum low density lipoprotein values of the experimental group and the control group. P values < 0.05 from the 1st week. See statistical differences in the two groups

### Testing of common carotid artery intima-media thickness (IMT) using ultrasound

We can observe statistical differences in the *t*-test between the two groups from the 5th week onwards, which suggests that the carotid artery IMT levels of the experimental group is higher than that of the control group from the 5th week (as seen in Table 3).

**Table 3:** Rabbit carotid artery intima-media (IMT) test results between the experimental group and the control group (mm)

Group	Week 1	Week 2	Week 3	Week 4	Week 5	Week 6	Week 7	Week 8	Week 9	Week 10
Exp.	0.179 ± 0.003	0.179 ± 0.003	0.181 ± 0.006	0.184 ± 0.006	0.203 ± 0.011	0.254 ± 0.018	0.337 ± 0.053	0.405 ± 0.069	0.448 ± 0.052	0.491 ± 0.043
Control	0.178 ± 0.004	0.178 ± 0.004	0.179 ± 0.007	0.180 ± 0.006	0.179 ± 0.003	0.188 ± 0.004	0.200 ± 0.005	0.202 ± 0.005	0.208 ± 0.006	0.214 ± 0.007
P value	0.516	0.516	0.537	0.248	0.012	0.001	0.001	0.002	0.001	0.001

Conduct *t* test on the rabbit carotid artery IMT values of the experimental group and the control group. P values < 0.05 from the 5th week. See statistical differences in the two groups

### WSS quantitative analysis of the common carotid arteries

#### WSS quantitative analysis of the common carotid arteries

Let us observe the *t*-test statistical differences between the two groups from the 1st week; it is seen that rabbit carotid arterial WSS of the experimental group is lower than that of the control group from the 1st week (as seen in Table 4).

**Table 4:** Shear stress test results between the experimental group and the control group (dyne/cm^2^)

Group	Week 1	Week 2	Week 3	Week 4	Week 5	Week 6	Week 7	Week 8	Week 9	Week 10
Exp.	1.90 ± 0.31	1.64 ± 0.16	1.54 ± 0.16	1.42 ± 0.16	1.34 ± 0.16	1.30 ± 0.12	1.21 ± 0.11	1.17 ± 0.11	1.13 ± 0.12	0.97 ± 0.08
Control	2.54 ± 0.09	2.74 ± 0.05	2.61 ± 0.07	2.39 ± 0.08	2.89 ± 0.13	2.49 ± 0.03	2.49 ± 0.16	2.47 ± 0.05	2.51 ± 0.08	2.64 ± 0.10
P value	0.0002	0.0001	0.0001	0.0001	0.0001	0.0001	0.0001	0.0002	0.0003	0.0001

Conduct *t* test on the rabbit shear stress values of the experimental group and the control group. P values < 0.05 from the 1st week. See statistical differences in the two groups

#### The columnar analysis diagram of common carotid arterial WSS

**Fig. 3:**
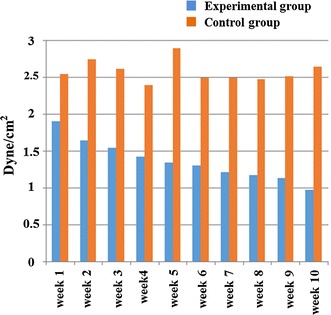
Dynamic variation diagram of the rabbit carotid arterial WSS value between the experimental and the control groups

In Fig. 3, it is shown that the carotid arterial WSS of the experimental rabbits changes and increases with time, while the carotid artery WSS of the control group has no obvious change.

#### Three-dimensional spatial distribution map of the carotid arterial WSS

**Fig. 4:**
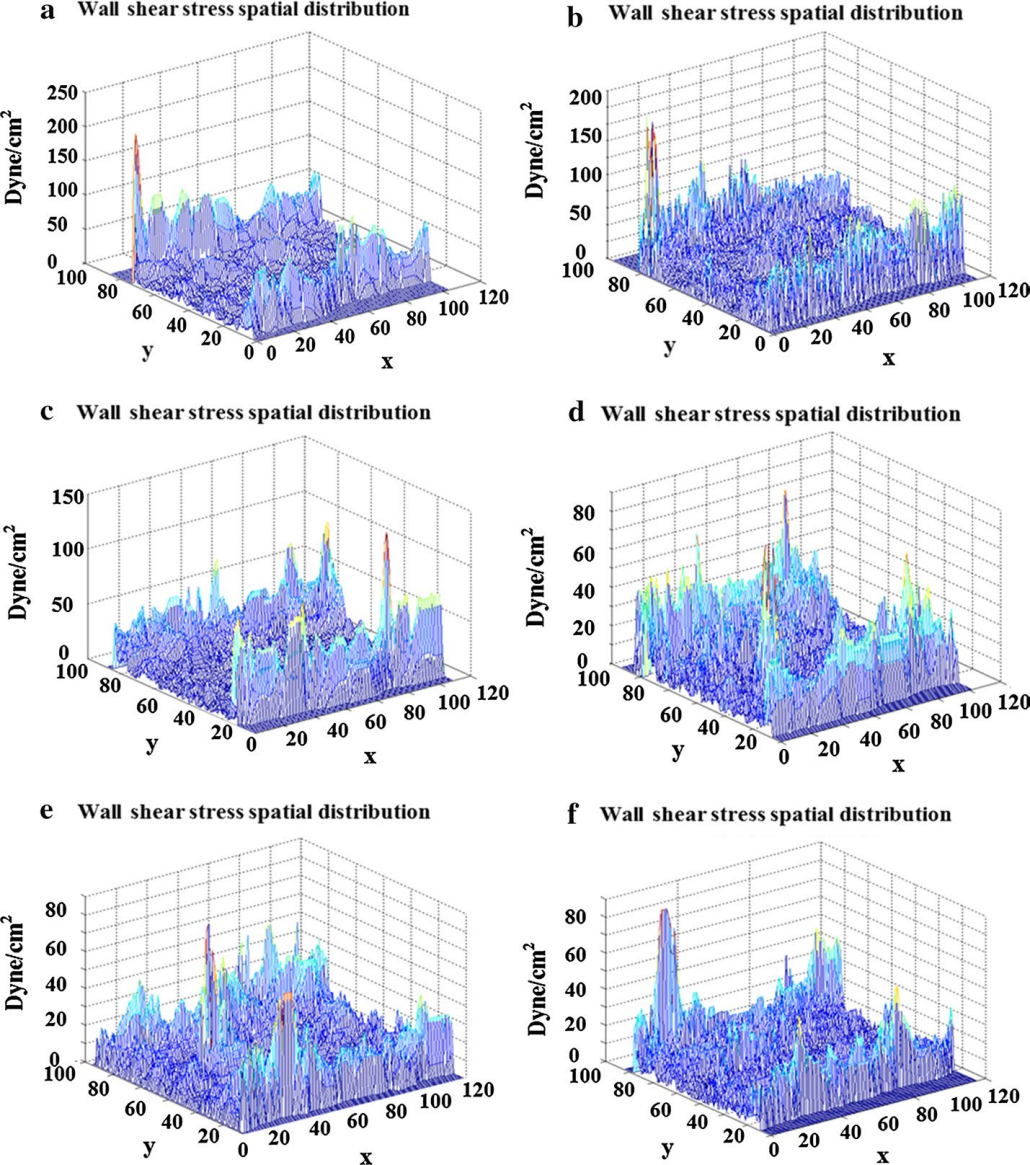
Rabbit common carotid arterial WSS 3d spatial distribution map. **a** A rabbit carotid arterial WSS of control group with range at 250; The carotid arterial WSS of experimental rabbits at 2 weeks (**b**), 4 weeks (**c**), 6 weeks (**d**), 8 weeks (**e**), and 10 weeks (**f**)

Figure 4 depicts the rabbit common carotid arterial WSS 3D spatial distribution map Therein, the *z* axis range represents the wall shear stress, wherein the highest peak is the maximum shear stress. The higher the degree of atherosclerosis, the lower is the WSS value. Figure 4 shows the range of the three-dimensional surface plots at 2, 4, 6, 8, and 10 weeks.

### Diagnostic efficiency of WSS

**Fig. 5:**
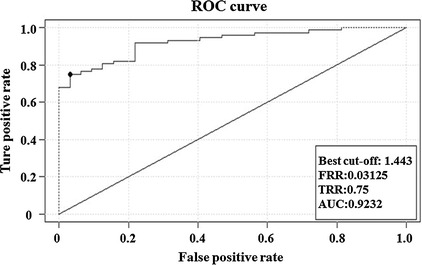
ROC curve of rabbit common carotid WSS based on mean value of shear stress at 1.443 dyne/cm^2^ demonstrating that the area under this curve is 0.9232 approx

**Fig. 6:**
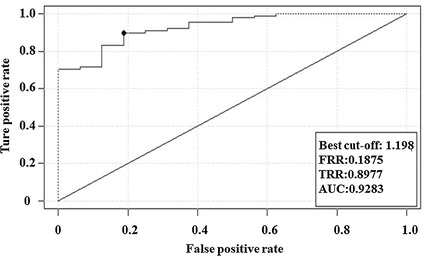
ROC curve of rabbit common carotid WSS based on mean value of shear stress at 1.198 dyne/cm^2^ demonstrating that the area under this curve is 0.9283 approx

When the mean value of shear stress is 1.443 dyne/cm^2^, the rabbit common carotid atherosclerosis fatty streaks sensitivity is 75% and the specificity is 96.9%. The area under the receiver operator characteristic (ROC) curve is 0.9232 (Fig. 5). For the mean value of shear stress at 1.198 dyne/cm^2^, the rabbit common carotid atherosclerosis fatty streaks sensitivity is 89.8% and the specificity is 81.3%. The area under the ROC curve is 0.9283 (Fig. 6). In summary, this ROC curve analysis has shown that when the mean value of shear stress is 1.198 dyne/cm^2^, the rabbit common carotid atherosclerosis fatty streaks sensitivity is 75% and the specificity is 96.9%. The area under the ROC curve is 0.9232 (Fig. 6). So the decreasing of WSS to 1.198 dyne/cm^2^ can be used as an indicator that rabbit common carotid artery atherosclerosis comes into the period of fatty streaks.

## Discussion

Atherosclerosis widely affects main and median arteries. The interaction between hemodynamics and the endothelium is an important determinant of cardiovascular function in human health, survival and morbidity. In hemodynamics, shear stress is the tangential stress acting on the endothelial surface. The endothelium is critical to cardiovascular health, as this layer of cells maintains anticoagulant properties and enables physiological control of vasoregulation and modulation of vascular permeability. Lipid (that mainly comprises cholesterol) deposits in the intima of main and median arteries. Smooth muscle cells and collagen fiber hyperplasia and atheromatous plaque formation causes different levels of luminal stenosis. It is a slow process of lesions, and the early stage of it is often neglected. Clinical ultrasound is commonly used to measure artery IMT so as to judge if there is atherosclerosis and then take the appropriate clinical intervention. But often the results are not obvious and the arterial injury cannot be reversed [18]. Therefore, IMT only has a limited sense on early diagnosis of atherosclerosis and predicting cardiovascular and cerebrovascular diseases.

In the process of atherosclerosis formation, hemodynamics alterations and pathological reconstitution occurs in the artery blood vessels. WSS is one of the important physical factors that affect the occurrence and development of atherosclerosis [19]. As a direct stress acting on arterial smooth muscle cells, it directly affects the arterial smooth muscle cells and arterial smooth muscle cell media, and thus indirectly affects other cellular components and structures of the arterial wall.

Among most blood vessels, the movement of blood flow is laminar. In theory, the velocity of fluid flow in blood vessels is different at all locations. The velocity of the outermost layer blood flow next to the vascular wall is zero, while the velocity of blood flow at the center of the cross section is the highest. This velocity gradient existing in a blood vessel tube is formed by the friction created by relative sliding between the flowing layers. The tangential friction between the two adjacent flowing layers in every unit area is the well-established blood flow parameter known as the Shear Stress (SS) [20].

**Fig. 7:**
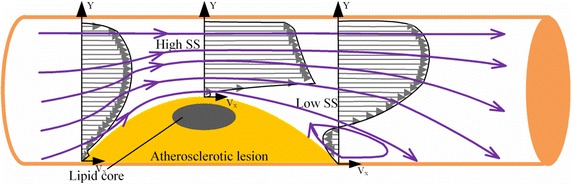
Flow separation and reversal in plaque vessel leads to low shear stress and further promotes formation and rupture of atherosclerotic plaque

Studies have confirmed that arterial WSS decrease is associated with the increase of blood lipid [21]. Arterial WSS decrease has close relationship with the increase of low density lipoprotein. Low WSS causes low density lipoprotein accumulation under the artery endothelium and induces formation of atherosclerosis. Figure 7 sums up how low WSS leads to the formation of atherosclerotic lesions and flow separations, resulting in pathologically disturbed flow [22]. WSS is different at different parts of the same blood vessel and atherosclerotic plaques are easier to be seen at the position with the lowest WSS. So the decreasing of WSS is a key step to cause atherosclerosis. Through the quantitative analysis of blood flow shear stress, this study has also proved that rabbit carotid arterial WSS values of the experimental group decrease when the blood lipid rises, which is statistically different from that of the control group (see Tables 1, 2, 3, 4).

## Conclusion

In this study, it is shown how WSS quantitative analysis can quickly assess the WSS of arterial blood flow, leading to atherosclerosis development. Implementing the Hagen–Poiseuille formula does not restrict us to factors such as blood flow, blood pressure, tube wall geometry, intima-media thickness, etc. We can employ it to quantitatively analyze the WSS of any point. Finally, it has the characteristics of being simple, fast and gives high accuracy prediction.

At the period of formation of atherosclerotic fatty streaks and early fibrous plaques, there are no clear clinical symptoms of stenosis caused by atherosclerosis, especially when it is the clinical precancerous lesion of atherosclerosis. At this stage, WSS is reduced, and has statistical significance when compared with the control group of rabbit animal models. This shows that WSS is closely related to atherosclerosis, and it can effectively be used as one of the effective parameters of occurrence and development of atherosclerosis.
